# Digital Simulation Improves, Maintains, and Helps Transfer Health-Care Providers' Neonatal Resuscitation Knowledge

**DOI:** 10.3389/fped.2020.599638

**Published:** 2021-01-18

**Authors:** Simran K. Ghoman, Maria Cutumisu, Georg M. Schmölzer

**Affiliations:** ^1^Neonatal Research Unit, Centre for the Studies of Asphyxia and Resuscitation, Royal Alexandra Hospital, Edmonton, AB, Canada; ^2^Department of Pediatrics, Faculty of Medicine and Dentistry, University of Alberta, Edmonton, AB, Canada; ^3^Department of Educational Psychology, Faculty of Education, University of Alberta, Edmonton, AB, Canada; ^4^Department of Computing Science, Faculty of Science, University of Alberta, Edmonton, AB, Canada

**Keywords:** neonatal resuscitation, simulation based education, digital simulation, healthcare education, simulation, table-top simulator, digital simulator

## Abstract

**Purpose:** To safely care for their newborn patients, health-care professionals (HCP) must undergo frequent training to improve and maintain neonatal resuscitation knowledge and skills. However, the current approach to neonatal resuscitation simulation training is time and resource-intensive, and often inaccessible. Digital neonatal resuscitation simulation may present a convenient alternative for more frequent training.

**Method:** Fifty neonatal HCPs participated in the study (44 female; 27 nurses, 3 nurse practitioners, 14 respiratory therapists, 6 doctors). This study was conducted at a tertiary perinatal center in Edmonton, Canada from April–August 2019, with 2-month (June–October 2019) and 5-month (September 2019–January 2020) follow-up. Neonatal HCPs were recruited by volunteer sampling to complete a demographic survey, pre-test (baseline knowledge), two digital simulation scenarios (intervention), and post-test (knowledge acquisition). Two months later, participants repeated the post-test (knowledge retention). Five months after the initial intervention, participants completed a post-test using a table-top simulation (knowledge transfer). Longitudinal analyses were used to compare participants' performance over time.

**Results:** Overall the proportion of correct performance increased: 21/50 (42%) passed the pre-test, 39/50 (78%) the post-test, 30/43 (70%) the 2-month post-test, and 32/40 (80%) the 5-month post-test. GLMM and GEE analyses revealed that performance on all post-tests was significantly better than the performance on the pre-test. Therefore, training with the RETAIN digital simulation effectively improves, maintains, and transfers HCPs' neonatal resuscitation knowledge.

**Conclusions:** Digital simulation improved, maintained, and helped transfer HCPs' neonatal resuscitation knowledge over time. Digital simulation presents a promising approach for frequent neonatal resuscitation training, particularly for distance-learning applications.

## Introduction

Each year, ~10% of infants worldwide need help to breathe at birth. To safely care for these newborn patients, health-care professionals (HCPs) must master their knowledge and decision-making skills outlined by the neonatal resuscitation algorithm. However, one million infants still die each year from asphyxia at birth (1). Alarmingly, half of these deaths are caused by deficiencies in HCPs' competence to safely provide care (2). To improve these deficiencies, guidelines recommended simulation-based education to target this root cause of mortality and prepare HCPs to safely provide neonatal resuscitative care (2, 3).

However, despite widespread uptake of the simulation-based Neonatal Resuscitation Program (NRP), HCPs remain dangerously underprepared for clinical neonatal resuscitation events due to decay in clinical skills after training (3). One reason for this persistent problem is that current simulation-based education is resource demanding, requiring lab space, specialized equipment, and trained instructors (4). Therefore, frequent simulation-based education opportunities are overall inaccessible by most HCPs (5).

Alternative approaches to traditional in-person simulation, such as digital simulation, may provide a solution. Digital simulation offers a scalable online platform for learners to readily access educational content and achieve many of the learning objectives of traditional simulation-based education, particularly for training HCPs' knowledge, including the recall of the correct sequence of steps for a clinical procedure (6). Digital simulation has been used successfully for a wide range of health-care education needs, such as surgical skills training (7) and disaster preparedness (8). Therefore, digital simulation may also offer a convenient approach to improve HCPs' competence to provide neonatal resuscitation care by improving knowledge and correct recall of the neonatal resuscitation algorithm (6).

This study aimed to examine if training with a digital simulation improves HCPs' neonatal resuscitation knowledge over time. This study also aimed to measure HCPs' knowledge transfer from the digital simulation. We hypothesized that HCPs would experience short- and long-term knowledge improvement of the correct steps of neonatal resuscitation after training with the digital simulation and transfer this knowledge into a novel learning environment.

## Methods

### Participant Recruitment

Neonatal HCPs were recruited by voluntary sample from the Royal Alexandra Hospital Neonatal Intensive Care Unit (NICU), Edmonton, Canada, a tertiary perinatal center delivering over 6,500 infants annually. Recruited participants included registered nurses, respiratory therapists, doctors, and neonatal nurse practitioners. The demographic characteristics (e.g., clinical position, gender, etc.) of the study sample was representative of the HCP population within the NICU. The study was performed at the bedside in the NICU and was approved by the Human Research Ethics Board at the University of Alberta, Edmonton, Canada (Pro00081221). Written informed consent was obtained from HCPs prior to participation.

### RETAIN Digital Simulator

RETAIN (REsuscitation TrAINing; RETAIN Labs Medical Inc. Edmonton, Canada; https://www.retainlabsmedical.com/index.html) is a digital simulator for HCPs to practice their knowledge and decision-making of neonatal resuscitation (9–12). In RETAIN, users are presented with scenarios of varying difficulty and medical conditions. After reviewing the case history and gathering a virtual team of HCPs, users make assessments and perform actions in response to continuous visual and cardiorespiratory feedback from the infant. Appropriate actions improve the infant's health, whereas inappropriate actions cause the infant's health to worsen. To improve realism, information from the delivery room was incorporated, including the average time taken to complete each available action (e.g., the time needed to attach a pulse oximeter), and all scenarios are based on information recorded during real deliveries from the Royal Alexandra Hospital (Edmonton, Canada) (11).

### Participants and Procedure

**Figure 1:**
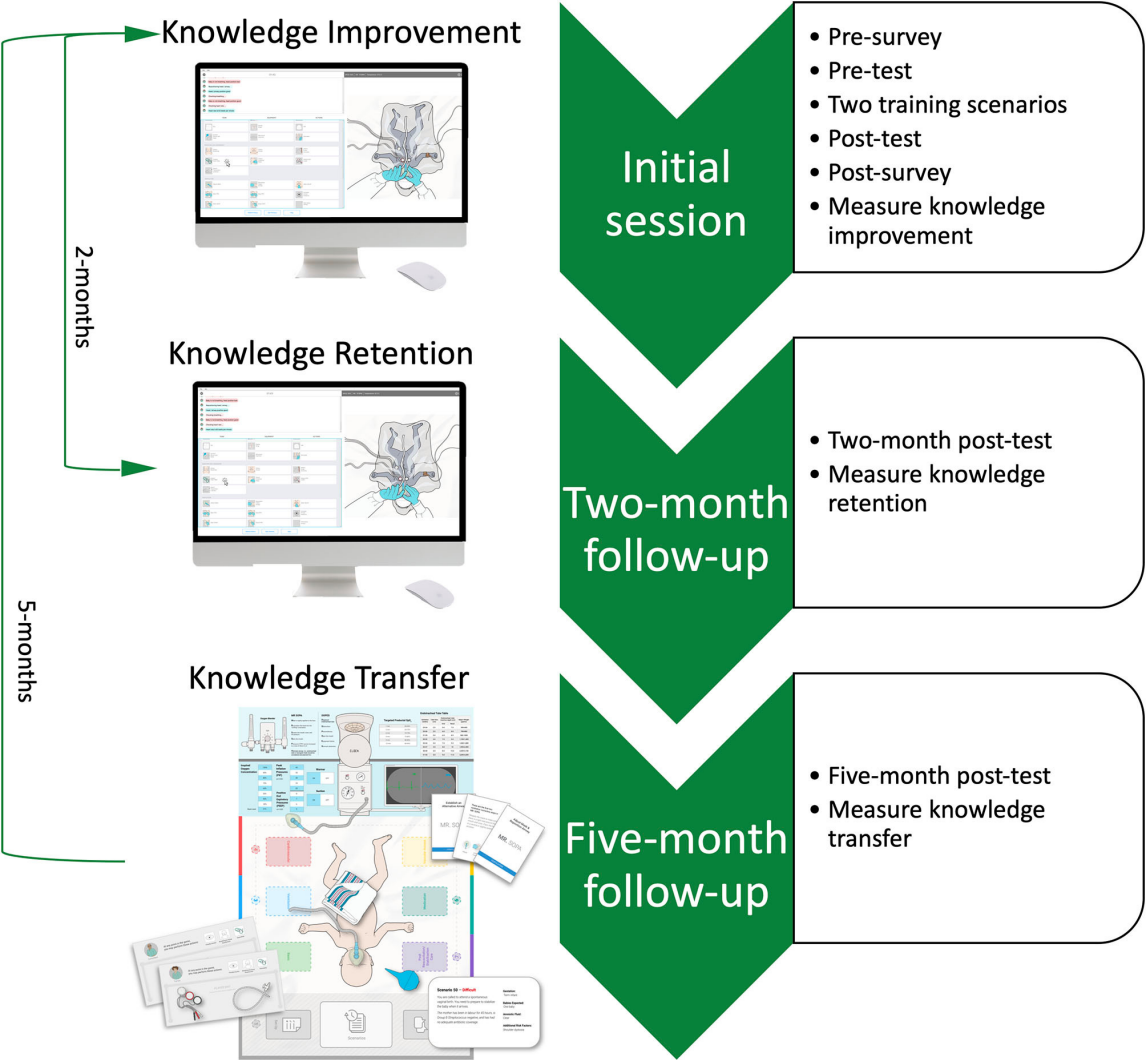
Schematic of study design.

Participants were *n* = 50 neonatal HCPs (44 females, 6 males; 27 registered nurses, 3 nurse practitioners, 14 respiratory therapists, and 6 doctors) who completed NRP-recertification within the last 24-months. The study consisted of four different assessments administered over three different timepoints: an initial session (including a pre-test and a post-test), a 2-month follow-up session, and a 5-month follow-up session (Figure 1).

#### Initial Session

All participants completed a demographic survey, including their clinical position, years of neonatal experience, and time elapsed since last NRP certification course. Following the demographic survey, participants were allowed to familiarize themselves with the digital simulator interface and completed a guided tutorial of a neonatal resuscitation scenario.

Once familiarized, participants completed the *pre-test* to measure participants' baseline knowledge. The *pre-test* consisted of a neonatal resuscitation simulation scenario requiring correct escalation through the NRP algorithm up to chest compressions to successfully stabilize the infant (the complete assessment is available in Appendix 1). There was no formal feedback provided to participants about their performance during or after the *pre-test*.

After the *pre-test*, participants completed two digital simulation training scenarios. The training scenarios were followed by a *post-test* to measure changes in participants' knowledge after training with the RETAIN digital simulator. The *post-test* scenario required correct escalation through the NRP algorithm up to chest compressions (the complete assessment is available in Appendix 1).

#### Two-Month Follow-Up

Two-months after the initial session, participants' long-term knowledge retention was assessed using a *2-month follow-up post-test*. The *2-month follow-up* scenario required correct escalation through the NRP algorithm up to chest compressions (the complete assessment is available in Appendix 1).

#### Five-Month Follow-Up

Five-months after the initial session, participants' knowledge transfer to a new learning environment was examined with a *5-month follow-up post-test* using a low-fidelity table-top simulation (Figure 1). In the table-top simulator, players use action cards (e.g., initiate positive pressure ventilation), equipment pieces (e.g., T-piece mask), and adjustable monitors (e.g., increase peak inspiratory pressure), to stabilize a low-fidelity simulated newborn infant presented on a 2D board. More information about the table-top simulator is presented in the literature (6, 9, 11). The action cards are identical to the action buttons presented in the digital simulator. Throughout the scenario, players receive standardized feedback (i.e., heart rate, oxygen saturation, etc.) from a facilitator (trained researcher) in response to their actions. The *5-month follow-up* scenario required correct escalation through the NRP algorithm up to administering one dose of epinephrine *via* the umbilical venous catheter (the complete assessment is available in Appendix 1).

### Measures

All scenarios were scored based on the 7th edition NRP guidelines (3). Participants' time-stamped actions (i.e., mouse input and keystrokes) during each scenario were recorded within the digital simulator. During the table-top simulation scenario, participants' actions were recorded by a trained researcher (SKG) using a sequential checklist (Appendix 1).

Participants completed all scenarios individually without assistance. Feedback was only provided through visual and cardiorespiratory assessment of the infant throughout the scenario (e.g., heart rate *via* electrocardiogram monitor, oxygen saturation *via* pulse oximeter). Depending on the steps taken by the participant, the infant's health improved, worsened, or remained unchanged (i.e., heart rate, oxygen saturation, etc.). If all steps of the NRP algorithm were performed correctly, the infant's health would improve, the infant would stabilize, and the scenario would end. If participants completed any step of the NRP algorithm incorrectly, the infant would fail to stabilize, and participants would exit the scenario.

The *pre-test, post-test, 2-month follow-up*, and *5-month follow-up* measures represented the cumulative score across all actions, interventions, or tasks initiated by participants during the assessment (Appendix 1). For each measure, participants were assigned a score of either pass or fail. A passing score represented 100% correct adherence to the NRP guidelines (3). A failing score represented <100% correct adherence to the NRP guidelines (3). Because deviation from the algorithm resulted in deterioration of the simulated infant's health, and this deterioration may have prompted participants to deviate further from the sequence expected by the digital simulator, a binary pass or fail outcome was used. The *pre-test, post-test, 2-month post-test*, and *5-month post-test* shared common actions across all four scenarios (NRP algorithm up to 1 min of chest compressions). However, the *5-month post-test* to measure knowledge transfer included two additional actions (obtaining vascular access and administering one dose of epinephrine).

Participants had no access to the RETAIN simulator in between data collection timepoints (digital simulator and table-top simulator were kept locked in the NICU research office), nor had any of the participants previously used the digital nor tabletop simulator described in the present study. The only training scenarios for participants to practice their neonatal resuscitation knowledge occurred during the initial session (two digital simulation scenarios administered). In contrast, the *pre-test, post-test, 2-month post-test*, and *5-month post-test* were assessment scenarios. The RETAIN table-top simulation was previously reported as a method for summative assessment of neonatal resuscitation providers (9).

### Analyses Plan

Statistical analyses were performed using RStudio AGPL v3 Desktop Open Source Edition (RStudio Inc., Boston, MA) with R version 4.0.1 (13). We conducted descriptive analyses to ascertain whether the performance outcome changed over time. Correlations between performance scores at different timepoints were explored to ascertain whether a possible dependency among the multiple measurements across the longitudinal data changed over time. Following this, we conducted repeated-measure analyses with binary outcomes (i.e., solving the test scenario correctly or incorrectly): generalized linear mixed models (GLMMs) and generalized estimating equations (GEEs). Data are presented as median [interquartile range (IQR)] for non-normal or mean [standard deviation (SD)] for normally distributed continuous variables.

## Results

The number of months since participants completed their last NRP course was median (IQR) 9 (5–12) months, and number of years of clinical neonatal experience was median (IQR) 9 (6.4–15.2) years. All participants completed the initial session, consisting of the *pre-test* (baseline assessment), training scenarios, and *post-test* (immediate knowledge improvement). Forty-three participants (*n* = 3 job relocation, *n* = 2 maternity leave, *n* = 3 unavailable) completed the 2-month follow-up session, consisting of the *2-month post-test* (long-term knowledge retention). Forty participants (*n* = 1 maternity leave, *n* = 2 unavailable) completed the 5-month follow-up session, consisting of the *5-month post-test* (knowledge transfer).

### Descriptive Statistics

We reported an overall increase in the proportion of correct performance across tests: 21 out of 50 participants passed the *pre-test* (42%), 39 of 50 participants passed the *post-test* (78%), 30 of 43 participants passed the *2-month post-test* (70%), and 32 of 40 passed the *5-month post-test* (80%). The distributions of the performance at each timepoint were explored using violin plots from the *ggplot2* package (available in Appendix 2).

Although performance slightly declined at the 2-month testing point (as expected with the passage of time), mean performance after 5-months was double the mean performance on the *pre-test*, suggesting that learning was successfully sustained and transferred. Note that the *5-month post-test* was a more difficult scenario than that of the previous tests.

### Tests of Association

Pearson correlation analyses revealed that the *pre-test* was significantly associated with the *5-month post-test* (*r* = 0.43, *p* < 0.05). Also, the association between the *pre-test* and the immediate *post-test* did not reach significance (*r* = 0.26, *p* > 0.05). Results were identical for the Spearman correlation as well.

### Repeated-Measure Analysis

#### Generalized Linear Mixed-Effects Models (GLMM)

GLMM models with dichotomous longitudinal outcomes were fit using the *glmer* function of the *lme4* package in R (14) by maximum likelihood (Laplace Approximation), with the *family* argument set to *binomial*, and the *logit* link function. The results of the model fit are summarized in Table 1. Positive fixed effects for time suggest that performance on all *post-tests* is significantly better than the performance on the *pre-tests* (see the β_time1_, β_time2_, and β_time3_ estimates).

**Table 1:** GLMM model fit for performance over time.

	**Random Intercept** **+** **Time GLMM**			
	**Estimate**	**SE**	***p-value***	**95% Confidence Interval**
(Intercept)	−0.389	0.341	0.25	[−1.095, 0.273]
Time 1	1.876	0.517	***	[0.915, 2.961]
Time 2	1.360	0.497	**	[0.422, 2.386]
Time 3	2.007	0.563	***	[0.968, 3.193]
AIC/AICc	220			
BIC	236			
logLik	−105			
Deviance	210			
df.resid	178			

*** *p < 0.001*,

** *p < 0.01; SE, Standard error; GLMM, Generalized linear mixed model; BIC, Bayesian Information Criterion; AIC, Akaike Information Criterion; AICc, AIC correction for small sample sizes*.

**Figure 2:**
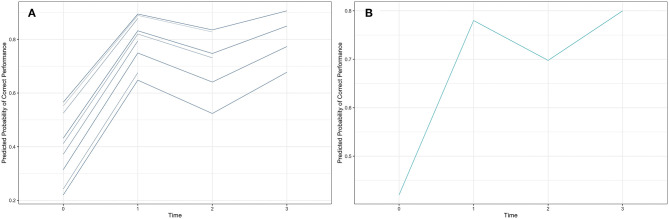
The visualization of the predicted probabilities of correct performance at each testing time point (0: pre-test, 1: post-test, 2: 2-month post-test, and 3: 5-month post-test), for the GLMM random-intercept with time model **(A)** and the GEE model **(B)**.

The *ggplot* function from the *ggplot2* R package was used to plot the results of the prediction expressed in probabilities shown in Figures 2A,B, which displays the individual participant-predicted trajectories based on the final GLMM model. The results observe that participants vary in their starting performance on the *pre-test*, captured by the differences in starting points displayed on the left side of Figures 2A,B, and that some participants did not complete one or two of the last two *post-tests*. The variability in the intercepts modeled with the random-intercept term indicates that some groups of participants had high model implied probabilities of performing correctly throughout all testing stages, whereas others had lower probabilities throughout all of the four testing points.

#### Generalized Estimating Equations (GEE)

The *geeglm* function of the *geepack* package in R, with *binomial* distribution and *logit* link function, was used to fit the GEE models that estimate the population aggregate (average) trend directly to make inferences about the population rather than about an individual. The *QIC* function of the *MuMIn* package in R was used to compute the quasi-information criterion (QIC) statistic that guides model selection (where a smaller value indicates a better fit). The GEE model selection is detailed in Appendix 2. As in the case of the GLMM model, the results of the GEE model fit show an increasing average trend in performance over time, as illustrated in Table 2 and Figure 2.

**Table 2:** GEE model fit for performance over time.

	**Random Intercept** **+** **Time GEE**			
**Term**	**Estimate**	**SE**	***p-value***	**95% Confidence Interval**
(Intercept)	−0.323	0.287	0.26	[−0.884, 0.239]
Time 1	1.588	0.446	***	[0.715, 2.46]
Time 2	1.159	0.439	**	[0.299, 2.02]
Time 3	1.709	0.488	***	[0.752, 2.67]
QIC	221			

*** *p < 0.001*,

** *p < 0.01; SE, Standard error; GEE, Generalized estimating equation; QIC, Quasi-Information Criterion*.

## Discussion

Lifetime risk of mortality is the highest on the day of birth (15). Birth asphyxia is the third biggest cause of neonatal mortality, claiming the lives of one million infants each year (16). Effective resuscitation helps reduce neonatal mortality (17); therefore, effective resuscitation training for HCPs will help more infants survive their first day after birth. While there is a clearly defined algorithm describing the steps of neonatal resuscitation, two-thirds of infant mortality during neonatal resuscitation is caused by deficiencies in HCPs' competence to safely provide care (2, 3). Neonatal resuscitation is a stressful, high-acuity medical intervention; therefore, deviations from the algorithm are dangerously common (18, 19).

Simulation-based education is recommended to address these deficiencies by replicating a real-world clinical situation, while simultaneously maintaining patient safety (20). Simulation-based education improves HCPs' neonatal resuscitation competence (21); however, frequent training sessions are needed to retain competence (22). Unfortunately, the current approach to simulation-based education is limited by intense time (e.g., scheduling sessions, developing scenarios) and resource (e.g., simulation lab, manikin, trained instructor) requirements. Therefore, frequent training sessions are often overall inaccessible.

In contrast, digital simulation presents a promising alternative to overcome traditional time and resource limitations of neonatal resuscitation simulation. Digital simulation may offer a convenient solution for neonatal resuscitation providers to access more frequent training to foster sustained learning (23). Several digital simulators have been developed for neonatal resuscitation, including RETAIN (6, 11). The RETAIN digital simulator presents HCPs with iterative resuscitation scenarios based on real deliveries recorded at a tertiary perinatal center. We sought to understand if training with the RETAIN digital simulator improved HCPs' knowledge of the correct steps of neonatal resuscitation, and if the improvement was sustained long-term.

Our participants were experienced neonatal HCPs recruited from a tertiary perinatal care center (median of 9 years of clinical experience). When compared to their baseline scores, we observed that HCPs significantly improved their performance after training with the RETAIN digital simulator, although there was some variation in the *pre-test*, likely due to the variety and size of the sample. Therefore, a single session with the RETAIN digital simulator immediately improved performance demonstrated by highly experienced neonatal resuscitation providers. Furthermore, HCPs' improvements were sustained 2-months after training. The proportion of correct performance tended to increase over time, with only small variations. The median of the *pre-test* was smaller than the mean, suggesting that there are a few participants who performed very well on the *pre-test* and pulled the mean of the group above the median. Performance on the *pre-test* tended to be slightly poor, but the trend was reversed for all three *post-tests*, more so for the immediate *post-test* and for the *5-month post-test*. All *post-tests* had similar variation, higher than that of the *pre-test*. Therefore, training with the RETAIN digital simulation resulted in HCPs' long-term knowledge retention.

We expected the correlation between performance scores at different timepoints to decline. That was the case between the *pre-test* and the *2-month post-test*. However, the strongest correlation found was between the first (*pre-test*) and the last (*5-month post-test*) timepoints. This result may be explained by the different nature of the *5-month post-test*; however, this result is promising, considering the *5-month post-test* administered a more complex scenario compared to previous tests.

To measure knowledge transfer, we used a table-top simulator previously reported as a valid summative assessment tool to evaluate neonatal resuscitation performance (9). Knowledge transfer from one situation to another constitutes the essence of analogical thinking but rarely happens spontaneously because it hinges on the learner's deep understanding of a concept before being able to recognize its general form across widely different instances (24). However, knowledge transfer is important for developing good problem-solving and decision-making skills because it helps learners to apply their knowledge to unfamiliar learning contexts, when they need to make difficult decisions on their own (25). The results from this study indicate that RETAIN effectively improves, maintains, and transfers HCPs' neonatal resuscitation knowledge.

The RETAIN digital simulator provides neonatal resuscitation scenarios of varying difficulty and patient risk factors, accessible by HCPs anywhere and anytime (6, 11). Digital simulation could be used to improve access to neonatal resuscitation training for HCPs from low-resource or rural health-care sites, as well as allow for more frequent and convenient training for HCPs at high-resource urban sites. Interestingly, evidence-based digital simulation may also help solve the emerging urgent need for remote health-care education strategies. In response to the recent coronavirus disease 2019 (COVID-19) pandemic, simulation-based education has faced significant interruptions that will likely continue for the foreseeable future. Many simulation centers have closed in an effort to maintain social distancing, preserve personal protective equipment, redistribute resources, and transition to clinical space. Health-care education, training, certification, and licensure has either been postponed indefinitely or transitioned to remote delivery. Many institutions have relied on remote training methods like online lectures or textbooks, which often constitute passive learning activities and may lack clinical relevance.

In contrast, digital simulation offers a feasible approach toward active remote learning, which may be readily taken up by neonatal resuscitation providers during COVID-19. Widespread implementation of digital simulation can provide a practical tool for health-care institutions to transition to effective and clinically relevant remote learning strategies. In consideration of current public health challenges, continuation of health-care education is essential to improve HCP performance so they may continue to provide safe and effective health care for their patients during these unprecedented times.

### Limitations

The RETAIN digital simulator was not designed with the goal of improving psychomotor practical skills. Therefore, the current version of the RETAIN digital simulator should be used to reinforce rather than replace traditional simulation-based education. It is not clear whether improved performance on a digital simulator indicates competence in providing neonatal resuscitation care. Other limitations of the study include convenience sampling, lack of random assignment and control group. However, our attrition rate was reasonable, considering participants were HCPs on a busy intensive-care unit, which made retesting difficult. Further, there was no difference between *pre-test* scores of HCPs who dropped out compared to those who remained. Additional studies are needed to compare digital simulation to other remote learning methods and to measure changes in HCPs' performance in the delivery room after training with digital simulation.

## Conclusions

After using the RETAIN digital simulator, HCPs demonstrated improved adherence to the neonatal resuscitation algorithm, which was sustained 2- and 5-months after the initial training. HCPs also successfully demonstrated knowledge transfer to a new learning environment. The RETAIN digital simulator may be useful to supplement formal neonatal resuscitation education, with potential application for remote learning during the COVID-19 pandemic.

## Data Availability Statement

The original contributions presented in the study are included in the article/Supplementary Material, further inquiries can be directed to the corresponding author/s.

## Ethics Statement

The study was performed at the bedside in the NICU and was approved by the Human Research Ethics Board at the University of Alberta, Edmonton, Canada (Pro00081221). Written informed consent was obtained from HCPs prior to participation. The patients/participants provided their written informed consent to participate in this study.

## Author Contributions

GS attests that all listed authors meet authorship criteria and that no others meeting the criteria have been omitted. SG, MC, and GS conceptualized and designed the study, designed the data collection instruments, collected data, carried out the analyses, and interpretation, drafted the initial manuscript, and critically reviewed and revised the manuscript. All authors approved the final manuscript as submitted and agreed to be accountable for all aspects of the work.

## Conflict of Interest

GS has registered the RETAIN table-top simulator (Tech ID 2017083) and the RETAIN digital simulator under Canadian copyright (Tech ID – 2017086) and is an owner of RETAIN Labs Medical Inc., Edmonton, Canada (https://www.retainlabsmedical.com/index.html). The remaining authors declare that the research was conducted in the absence of any commercial or financial relationships that could be construed as a potential conflict of interest.

## Abbreviations

COVID-19: Coronavirus disease 2019
GEE: Generalized estimating equation
GLMM: Generalized linear mixed model
HCP: Healthcare professional
IQR: Interquartile range
NICU: Neonatal Intensive Care Unit
NRP: Neonatal Resuscitation Program
QIC: Quasi-information criterion
RETAIN: REsuscitation TrAINing
SD: Standard deviation.
