# Supplementary material for: Digital Simulation Improves, Maintains, and Helps Transfer Health-Care Providers' Neonatal Resuscitation Knowledge
Source: Front Pediatr. 2021 Jan 18;8:599638. doi: 10.3389/fped.2020.599638 (PMC7848194; doi:10.3389/fped.2020.599638)
Supplement: Supplementary file 2 [file Data_Sheet_2.docx]

Supplementary Material

# Appendix 1

## Pre-test, post-test, and 2-month post-test scenario

1. Prepare for the delivery. Assign roles, check equipment, set ventilation device, gather supplies, call for assistance, and don personal protective equipment. Review the case history (term baby, clear fluid, and fetal bradycardia for the last 3 minutes). Discuss the plan for cord management with the obstetrician.
2. The baby has been born. Immediate cord clamping due to the baby being apneic and having poor muscle tone.
3. Complete initial assessment. Visual assessment reveals the baby has no muscle tone. Breathing and airway assessment reveals the baby is apneic. After auscultation, the heart rate is 40 beats per minute.
4. Complete basic interventions. Dry and maintain temperature; tactile stimulation, measure oxygen saturation, measure heart rate, and measure temperature. The heart rate is 40 beats per minute.
5. Initiate ventilation. After initiating positive pressure ventilation, heart rate is 40 beats per minute after 15 seconds, with no chest rise. Begin ventilation corrective steps. Adjust mask and reposition airway. The heart rate is 40 beats per minute. Suction the airway and open the mouth. The heart rate is 40 beats per minute. Adjust ventilation pressure. Heart rate is 40 beats per minute. Increase inspired oxygen concentration (or earlier).
6. Establish an alternative airway and confirm correct tube placement. The first attempt to establish an alternative airway is unsuccessful. Remove device, resume positive pressure ventilation, and repeat insertion. The second attempt to establish an alternative airway is successful. The heart rate is 40 beats per minute, and there is chest rise, misting of the tube, and end-tidal CO_2_. Continue positive pressure ventilation.
7. Initiate cardiovascular interventions. Increase oxygen concentration to 100% if not already. Give chest compressions. After 60 seconds of chest compressions, the heart rate is 70 beats per minute. Stop chest compressions and continue positive pressure ventilation. Adjust inspired oxygen concentration to maintain oxygen saturation according to local hospital policy.
8. Post-resuscitation. Ten minutes after birth, the baby is stabilized. The heart rate is 141 beats per minute and the oxygen saturation is 90%. Prepare to admit the baby to the NICU. Debrief.

## 5-month post-test scenario

1. Prepare for the delivery. Assign roles, check equipment, set ventilation device, gather supplies, call for assistance, and don personal protective equipment. Review the case history. Discuss the plan for cord management with the obstetrician.
2. The baby has been born. Immediate cord clamping due to the baby being apneic and having poor muscle tone. Visual assessment reveals thick meconium.
3. Complete initial assessment. Visual assessment reveals the baby has no muscle tone. Breathing and airway assessment reveals the baby is apneic, and the airways are blocked with meconium. After auscultation, the heart rate is 34 beats per minute.
4. Complete basic interventions. Dry and maintain temperature; tactile stimulation (optional), suction airway, measure oxygen saturation, measure heart rate, and measure temperature. The heart rate is 37 beats per minute.
5. Initiate ventilation. After initiating positive pressure ventilation, heart rate is 36 beats per minute after 15 seconds, with no chest rise. Begin ventilation corrective steps. Adjust mask and reposition airway. The heart rate is 34 beats per minute. Suction the airway and open the mouth. The heart rate is 35 beats per minute. Adjust ventilation pressure. Heart rate is 33 beats per minute. Increase inspired oxygen concentration (or earlier).
6. Establish an alternative airway and confirm correct tube placement. The first attempt to establish an alternative airway is successful. The heart rate is 42 beats per minute, and there is chest rise, misting of the tube, and end-tidal CO_2_. Continue positive pressure ventilation.
7. Initiate cardiovascular interventions. Increase oxygen concentration to 100% if not already. Give chest compressions. After 60 seconds of chest compressions, the heart rate is 43 beats per minute.
8. Establish vascular access. Vascular access is successfully obtained. After 60 seconds of chest compressions, the heart rate is 49 beats per minute.
9. Administer medication. Continue chest compressions and administer the first dose of epinephrine. After 60 seconds of chest compressions and the first dose of epinephrine, the heart rate is 89 beats per minute. Stop chest compressions and continue positive pressure ventilation. Adjust inspired oxygen concentration to maintain oxygen saturation according to local hospital policy.
10. Post-resuscitation. Ten minutes after birth, the baby is stabilized. The heart rate is 141 beats per minute and the oxygen saturation is 97%. Prepare to admit the baby to the NICU. Debrief.

# Appendix 2

The GLMM and GEE models explore the log-odds of success (i.e., the outcome coded as 1), representing the probability ratio of the outcome 1 compared with the outcome 0, transformed by taking the log. Analyses were conducted with time as a factor to explore the differences among all the testing timepoints. The Pre-test was considered to be the reference group (i.e., coded as 0) for ease of interpretation and three dummy variables were used in the model fitting: time1, time2, and time3 (coded as 1, 2, and 3, respectively). This section includes the details regarding the construction of the GLMM and GEE models.

## GLMM models

First, we built a null GLMM model (a random-effect intercept-only model with no predictors), where participants were allowed their own intercept (i.e., their own unique performance starting point that deviates from the average intercept). Following that, we built a random-effect intercept model with time as a fixed-effect predictor variable (i.e., trend). The *bobyqa* control option was used to fit both models. A full random-coefficients model could not be built (i.e., performance ~ 1 + time + (1 + time | ID)), where ID represents a participant’s unique identifier, as the current data set was too small to allow a random slope (i.e., n = 40 complete observations at all four time points). This model would have allowed each individual to have their own regression model, such as their own performance trajectory (i.e., individual slopes that vary about the average time trend), and to model the dependency due to repeated measures more adequately (e.g., decreasing correlation as time lag increases).

An empirical model comparison using the *anova* function in R was conducted to help determine which of the models shows evidence of better fit. Also, the *aictab* function from the *AICcmodavg* package in R was employed for GLMM model selection, generating the AICc metric (i.e., the AIC correction for small sample sizes; a smaller value indicates better model fit). Regarding model fit, the Random-Intercept with Time model is an improvement over the fit of the Null GLMM model. Thus, the final GLMM model is a Random-Intercept model with time as a fixed effect: *glmer(performance ~ 1 + time + (1|ID), data = dataset_long, family = binomial, control=glmerControl(optimizer="bobyqa"))*, where ID represents a participant’s unique identifier.

The results of the GLMM model with time as a factor are shown in Table 1. The parameter estimates for the fixed effects (i.e., representing the average performance trajectory) are represented as parallel lines, because each participant is assumed to have the same slope.

## GEE models

Second, we built GEE models to estimate the population aggregate (average) trend directly to make inferences about the population rather than about an individual. Along with specifying the distribution (*binomial*) and link (*logit*) function, the residual covariance structure (i.e., working correlation structure matrix) is needed to build these models, as it accounts for the repeated-measures dependency. The following residual covariance structures were compared: independent (i.e., it assumes that all time points are independent of each other, which is uncommon for repeated measures data), exchangeable (i.e., compound symmetric; it assumes equal correlations between all timepoints), and AR1 (i.e., first-order autoregressive structure; it assumes a decreasing correlation as the time lag between observations increases, which is common for longitudinal data). This comparison shows that the three GEE models are equivalent, as all the working correlation matrices yielded similar fit (QIC = 221) and overall significance. Finally, the model using the AR1 structure was selected, as it allows correlations between time points to decay over time and the repeated measures were taken at approximately equally-spaced times (approximately every two months). The final GEE model was defined as: *geeglm(performance ~ 1 + time, id = ID, family = binomial, scale.fix = TRUE, corstr = "ar1", data = dataset_long)*, where ID represents a participant’s unique identifier. The results of the GEE model with time as a factor are shown in Table 2. The GEE model is illustrated in Figure 2, which highlights the marginal interpretation of the model, where individual trajectories are not generated for each individual.

## Distribution of performance

The distributions of the performance at each time point were explored using violin plots from the *ggplot2* package (Supplementary Figure 1).

## Supplementary Figures


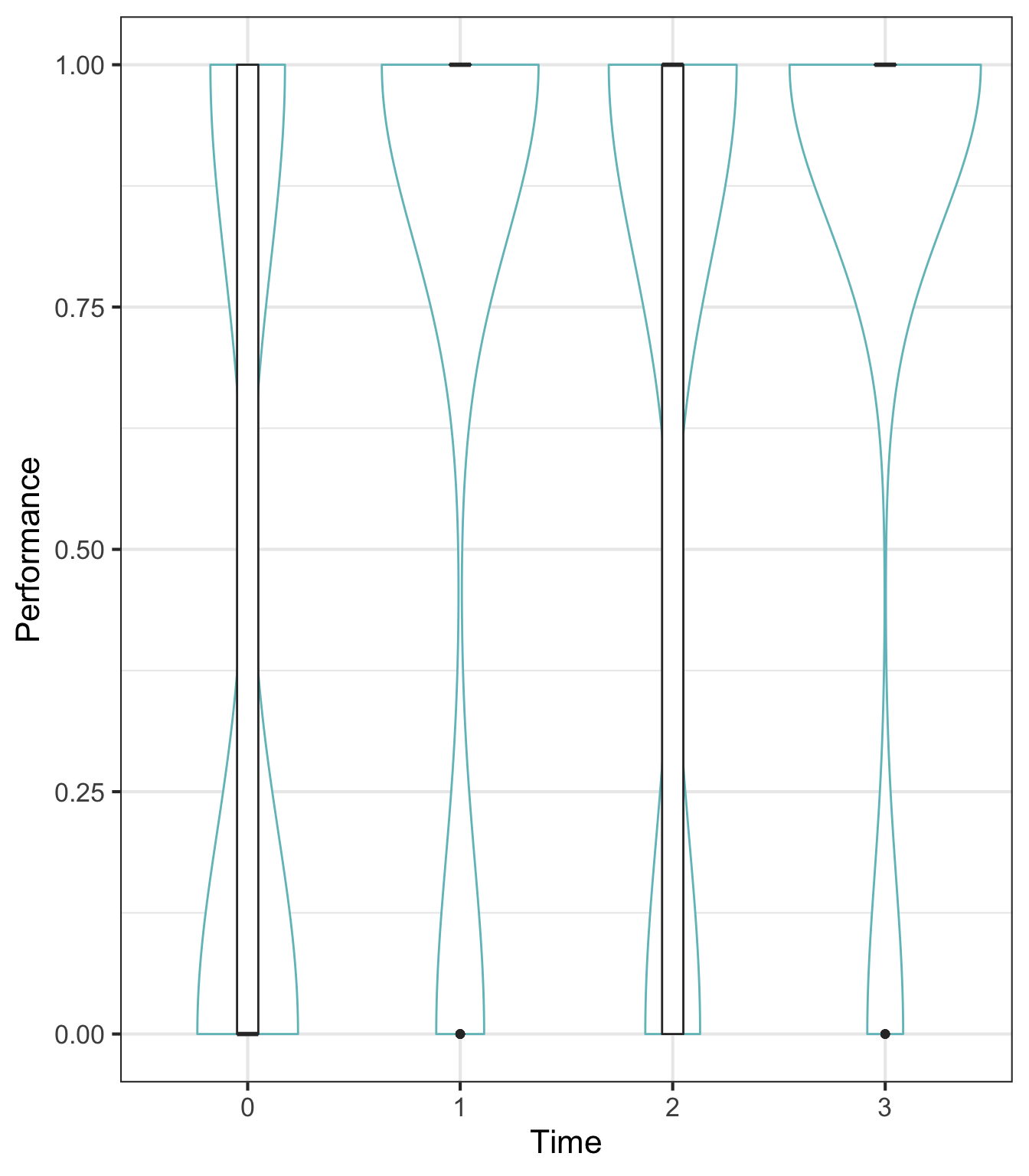


**Supplementary Figure 1.** Violin plots showing the performance distributions at the four timepoints, with median and quartile.
